# Visible blue light inhibits infection and replication of SARS-CoV-2 at doses that are well-tolerated by human respiratory tissue

**DOI:** 10.1038/s41598-021-99917-2

**Published:** 2021-10-18

**Authors:** Nathan Stasko, Jacob F. Kocher, Abigail Annas, Ibrahim Henson, Theresa S. Seitz, Joy M. Miller, Leslee Arwood, Rachel C. Roberts, Thomas M. Womble, Emily G. Keller, Soren Emerson, Michael Bergmann, Ashley N. Y. Sheesley, Rebecca J. Strong, Brett L. Hurst, David Emerson, E. Bart Tarbet, Shelton S. Bradrick, Adam S. Cockrell

**Affiliations:** 1 EmitBio Inc., 4222 Emperor Blvd, Suite 470, Durham, NC 27703 USA; 2 Division of Infectious Diseases, Surveillance and Diagnostics, MRIGlobal, Kansas City, MO 64110 USA; 3 Institute for Antiviral Research, Department of Animal, Dairy and Veterinary Sciences, Utah State University, Logan, UT 84321 USA

**Keywords:** Microbiology, Virology, SARS-CoV-2, Optics and photonics, Lasers, LEDs and light sources, Inorganic LEDs

## Abstract

The delivery of safe, visible wavelengths of light can be an effective, pathogen-agnostic, countermeasure that would expand the current portfolio of SARS-CoV-2 intervention strategies beyond the conventional approaches of vaccine, antibody, and antiviral therapeutics. Employing custom biological light units, that incorporate optically engineered light-emitting diode (LED) arrays, we harnessed monochromatic wavelengths of light for uniform delivery across biological surfaces. We demonstrated that primary 3D human tracheal/bronchial-derived epithelial tissues tolerated high doses of a narrow spectral band of visible light centered at a peak wavelength of 425 nm. We extended these studies to Vero E6 cells to understand how light may influence the viability of a mammalian cell line conventionally used for assaying SARS-CoV-2. The exposure of single-cell monolayers of Vero E6 cells to similar doses of 425 nm blue light resulted in viabilities that were dependent on dose and cell density. Doses of 425 nm blue light that are well-tolerated by Vero E6 cells also inhibited infection and replication of cell-associated SARS-CoV-2 by > 99% 24 h post-infection after a single five-minute light exposure. Moreover, the 425 nm blue light inactivated cell-free betacoronaviruses including SARS-CoV-1, MERS-CoV, and SARS-CoV-2 up to 99.99% in a dose-dependent manner. Importantly, clinically applicable doses of 425 nm blue light dramatically inhibited SARS-CoV-2 infection and replication in primary human 3D tracheal/bronchial tissue. Safe doses of visible light should be considered part of the strategic portfolio for the development of SARS-CoV-2 therapeutic countermeasures to mitigate coronavirus disease 2019 (COVID-19).

## Introduction

The SARS-CoV-2 pandemic continues to reap human and economic tolls around the world. As of July 2021, there are > 191.7 M cases and > 4.1 M deaths globally^1^. Notably, the United States is reporting the highest number of cases (> 34.1 M) and deaths (> 600 K) in the world^1^. Implementing public health measures (e.g. in-home quarantine and social distancing) that limit human-to-human transmission has proven to be insufficient to curb community transmission. The necessity to safely return individuals to the workplace and schools will likely require multiple, additional protective strategies beyond vaccination that enhance current public health measures. Accordingly, extending therapeutic intervention strategies beyond what is known today is critical.

To this end, development of SARS-CoV-2 therapeutic countermeasures is progressing at an unprecedented speed. The imperative need to mitigate acute respiratory distress symptoms associated with COVID-19 in patients has resulted in the emergency use authorization by the Food and Drug Administration (FDA) of the nucleoside analog Remdesivir, convalescent plasma, and antibody therapeutics^2–6^. In addition, the glucocorticoid, dexamethasone, was demonstrated to lower the mortality rate in individuals receiving either oxygen alone or in combination with mechanical ventilation support^7,8^. To curb the long timelines associated with clinical safety and efficacy trials for traditional drug therapeutics, researchers are also briskly working to evaluate existing FDA-approved drugs against SARS-CoV-2^9–11^. Although encouraging, many of the current strategies are limited in that they are SARS-CoV-2-specific and target the virus either exclusively outside (cell-free virus) or inside (cell-associated, replicating virus) the cell. Expanding the therapeutic arsenal beyond conventional strategies may expedite the availability of therapeutic countermeasures with antiviral properties that can inactivate both cell-free and cell-associated virus. One such expansion is light therapy.

Light therapy has the potential to inactivate both cell-free and cell-associated virus as a non-specific antiviral. Mitigating SARS-CoV-2 infection with light therapy requires a basic knowledge of which combination of wavelength and doses of light most effectively interferes with viral infection and replication while minimizing damage to host tissues and cells. A large body of literature demonstrates that ultraviolet light, predominantly ultraviolet C (UVC) at a peak wavelength of 254 nm, is highly effective at inactivating cell-free coronaviruses on surfaces, aerosolized, or in liquid^12–14^. UVC inactivates coronaviruses, as well as many other RNA and DNA viruses, through absorption of UVC photons by pyrimidines in the RNA, which lead to the formation of pyrimidine dimers that preclude replication of the coronavirus genome. While UVC is highly effective at inactivating coronaviruses, it can also be highly damaging to replicating mammalian cells. This damage can cause perturbations in genomic DNA that increase the risk of mutagenic events^15,16^. As such, viral inactivation with UV light is primarily limited to cell-free environmental applications such as disinfection of surfaces. Inactivating coronaviruses with safe, visible light (400–700 nm) would be a novel approach to interfering with SARS-CoV-2 infection and replication.

Photobiomodulation (PBM, otherwise known as light therapy) is the safe, low-energy, illumination of cells and tissues using LEDs or lasers with emission wavelengths in the visible or near-infrared light spectrum (400–1000 nm)^17^. Importantly, since the PBM therapeutic effect is driven by light’s interaction with photoacceptors within the biological system, PBM is not to be confused with photodynamic therapy (PDT) which employs the exogenous addition of photosensitizers or chemicals to induce reactive oxygen species. For example, the safe and effective use of blue light PBM in the 450–490 nm range was adopted for clinical use in the late 1960s to treat jaundice in neonates caused by hyperbilirubinemia and continues to be employed in hospitals today as a primary treatment for hyperbilirubinemia^18^. Studies also indicate that PBM with visible light may function to inactivate RNA and DNA viruses in vitro^19–22^. Several studies suggest that PBM therapy could be safely applied to the oral and nasal cavities with the goal to treat several illnesses^23–25^. While these studies are suggestive, a deeper exploration of the precise selection of monochromatic wavelengths of light applied over a range of optical doses to assess both safety and efficacy can broaden the scope of therapeutic applications in respiratory medicine. Specifically, in the delivery of light as a therapeutic, the product of radiant flux per unit area (Watts/cm^2^) and time in seconds defines the dose of light as optical energy per unit area in Joules/cm^2^.

There has been relatively little penetration of the PBM technique into clinical therapeutics for respiratory pathogens. This is due, in part, to the lack of a suitable illumination platform. This platform must be capable of applying precisely engineered light uniformly and repeatedly over a wide range of optical irradiances to biological samples while minimizing secondary effects such as optical heating. Further, the illumination platform must be agnostic to the type of biological samples to which the light is applied so that assays evaluating safety, efficacy, and biological mechanisms can be performed consistently. Finally, the illumination platform must be reproducible, transportable between labs, and fully compatible with biological safety practices to facilitate validation and reproducibility of results. Without such an illumination platform, the fundamental work needed to begin the journey to clinical application of PBM as a therapeutic for respiratory pathogens cannot be successful. Until now, an illumination platform such as that described has not been available. To evaluate the safety of light on cells and tissues in vitro as well as the efficacy of light in SARS-CoV-2 infectious assays, we developed a biological light unit (BLU) possessing the aforementioned properties. In the study presented here, we describe the first use of BLUs to deliver safe, visible wavelengths of blue light at selected energy densities to inactivate both cell-free and cell-associated SARS-CoV-2 in in vitro assays. Importantly, doses of blue light that effectively inactivate SARS-CoV-2 are well-tolerated by primary human tracheal/bronchial respiratory tissues.

## Results

### Biological light units for photobiomodulation

**Figure 1:**
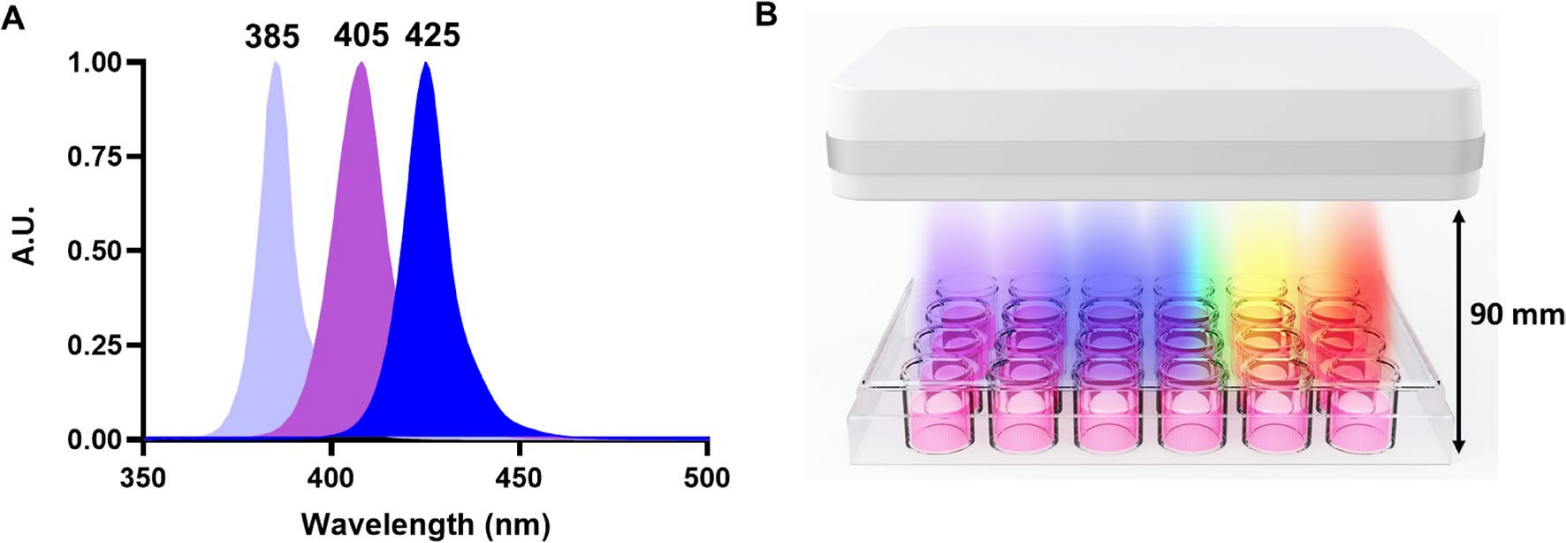
LED arrays for photobiomodulation. (**A**) Independent characterization of each LED array by measuring the spectral flux (385, 405, and 425 nm). Data is presented in arbitrary units (A.U.) for ease of comparison. (**B**) Illustration demonstrating the versatility of different LED arrays configured across the light spectrum to evaluate the photobiomodulation of cells and virus in a tissue culture system. Each LED array comprises a single narrow band wavelength of light that can be evenly distributed across a multi-well tissue culture plate, such that each replicate well receives the same dose of light.

A key element of the BLUs are optimized LED arrays characterized by narrow spectral emission profiles with peak wavelengths centered at 385 nm, 405 nm, and 425 nm (Fig. 1). When incorporated into the BLU, these LED arrays can illuminate the assay plates with calibrated, repeatable, and uniform doses of light so that illumination can occur reliably across many assays and in multiple laboratories. Each LED array was independently characterized by measuring the spectral flux (W/nm) relative to the wavelength (nm) (Fig. 1A). In our experiments, the peak wavelength of the 405 nm array is in the blue region of the visible spectrum, but 16.5% of the radiant flux extends into the UVA spectrum (< 400 nm), which is typical of LEDs with peak emission at 405 nm. The peak wavelength of the 385 nm LED array is clearly within the upper bounds of the UVA spectrum (315–400 nm) with 94% of the radiant flux in the UV spectrum. Conversely, 99% of the 425 nm array is found to be within the visible light spectrum (400–700 nm) when the full spectrum of emission is considered. Moreover, each LED array is verified to ensure that light is evenly distributed across multi-well tissue culture plates such that the biological test articles in each replicate well receives the same dose of light in each experiment. Similar attention to technical detail was given to the other critical design aspects of the BLU as was given to the LED arrays to ensure reproducibility and minimize illumination-induced temperature changes on the biological test articles and mimic the nominal distance to the oropharynx (Fig. 1B). Accordingly, the distance of the array from the biological test article, the optical irradiance, and the indicated doses (J/cm^2^) were carefully chosen to allow for consistent evaluation of visible light’s antiviral effect in physiologically relevant conditions.

### 425 nm blue light does not negatively impact human airway-derived 3D tissue models

**Figure 2:**
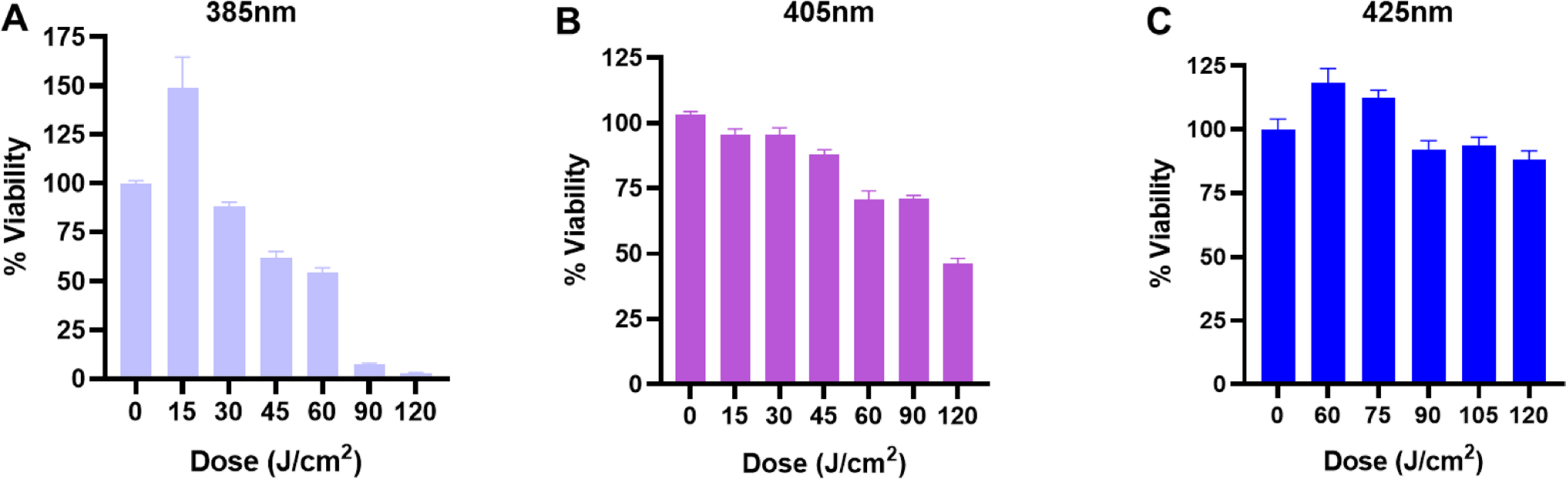
Primary 3D human tracheal/bronchial tissues exposed to light in a dose- and wavelength-dependent manner. Viability was assayed at 3 h post-exposure using the indicated doses of light at wavelengths of (**A**) 385 nm, (**B**) 405 nm, and (**c**) 425 nm. Data are represented as ± SEM.

Understanding how the target tissues in the upper airway tolerate blue light is central to the development of a light-derived antiviral approach to SARS-CoV-2. Initial evaluation using our BLUs was conducted on 3D tissue models developed from cells isolated from the bronchial/tracheal region of a single donor. The 3D EpiAirway tissue models are 3–4 cell layers thick comprising a mucociliary epithelium layer with a ciliated apical surface that has been validated to predict respiratory irritants after 3 h of exposure to a test article. To assess the wavelength and doses of blue light most tolerated by these tissues, replicate tissue samples were exposed to 385 nm, 405 nm, or 425 nm light at the indicated doses (Fig. 2). The percent viability was assessed using a well-established MTT cytotoxicity assay optimized by the manufacturer for the 3D EpiAirway tissue models. The viability of the tissues was clearly impacted in a wavelength- and dose-dependent manner (Fig. 2). Illumination with 385 nm light exhibited the most dramatic loss in viability with nearly a 50% decrease at a dose of 45 J/cm^2^ (Fig. 2A). Although less dramatic, 405 nm exhibited a dose-dependent decrease in viability with > 25% loss at 60 J/cm^2^ and a 50% loss at 120 J/cm^2^ (Fig. 2B). Importantly, the 425 nm light was well tolerated at doses of light as high as 120 J/cm^2^ (Fig. 2C). Longer wavelengths of visible light (425 nm) that do not have significant energy content in the UVA spectrum have minimal impact on the viability (< 20% loss at high doses) of primary human tissue derived from the upper respiratory tract. Based on these studies visible blue light at 425 nm was chosen for subsequent evaluation in the widely available Vero E6 cell line, conventionally used to evaluate SARS-CoV-2 infection and replication in vitro.

### 425 nm blue light impacts Vero E6 2D cell culture viability in a dose- and density-dependent manner

**Figure 3:**
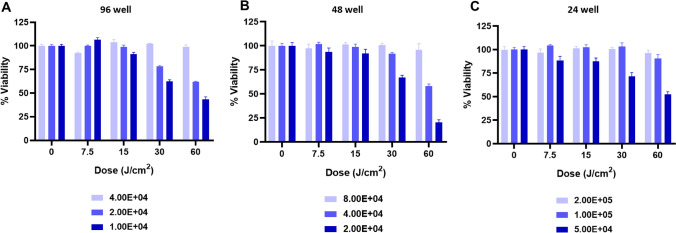
Vero E6 cell viability exposed to 425 nm light in a dose- and cell density-dependent manner. Vero E6 cells were seeded at different densities in (**A**) 96-well plate, (**B**) 48-well plate, and (**C**) 24-well plate and incubated overnight prior to illumination with 425 nm light at the indicated doses. Cell viability was determined 24 h post-illumination relative to the 0 J/cm^2^ dose. Data are represented as mean cell viabilities ± SEM.

Vero E6 cells are commonly used for preparing stocks, performing growth curves, and evaluating therapeutic countermeasures for SARS-CoV-2. Depending on the type of assay being performed it could be necessary to vary the seeding cell density and multi-well tissue culture plate format. Often, cell viability is evaluated to determine if the antiviral properties of a therapeutic can be parsed from potential therapeutic-induced cytotoxic effects. We sought to determine if cell density and multi-well plate format can influence cell viability upon exposure to 425 nm blue light. To effectively evaluate the cell viability, a cytotoxicity assay was optimized for use with Vero E6 cell densities up to 1 × 10^6^ cells/well on a 24-well plate (Supplementary Figure 1). The data in Supplementary Figure 1 show that 100 µL of CellTiter-Glo reagent is sufficient to quantitate the cell viability of Vero E6 cells (≤ 1 × 10^6^ cells) without reaching saturation of the chemiluminescent signal. Antiviral assays performed on 96-well plates are commonly evaluated at cell seeding densities of 1 × 10^4^ and 2 × 10^4^ cells incubated overnight. Under these conditions, we found that 425 nm blue light results in decreased viability (25–50%) at 30 and 60 J/cm^2^ by 24 h post-illumination, whereas a seeding density of 4 × 10^4^ cells tolerated high doses of light exposure (Fig. 3A). Unexpectedly, 4 × 10^4^ cells seeded on a 48-well plate was not tolerated (~ 50% reduction in viability with 60 J/cm^2^) compared to 8 × 10^4^ cells (Fig. 3B). These results demonstrated that the cell seeding density, and optical conditions of the multi-well plate, relative to the surface area of the culture well influences the susceptibility to the incident light. A similar trend was observed on the 24-well plate format, with cell densities > 10^5^ demonstrating acceptable viability at all doses tested (Fig. 3C). Higher Vero E6 seeding densities resulted in 100% cell confluence prior to illumination, exhibiting cell-to-cell contact that mimics the 3D EpiAirway models. Thus, high confluence Vero E6 cell monolayers readily tolerated 425 nm blue light as well as 3D EpiAirway tissue models.

### 425 nm blue light inhibits infection and replication of cell-associated SARS-CoV-2

**Figure 4:**
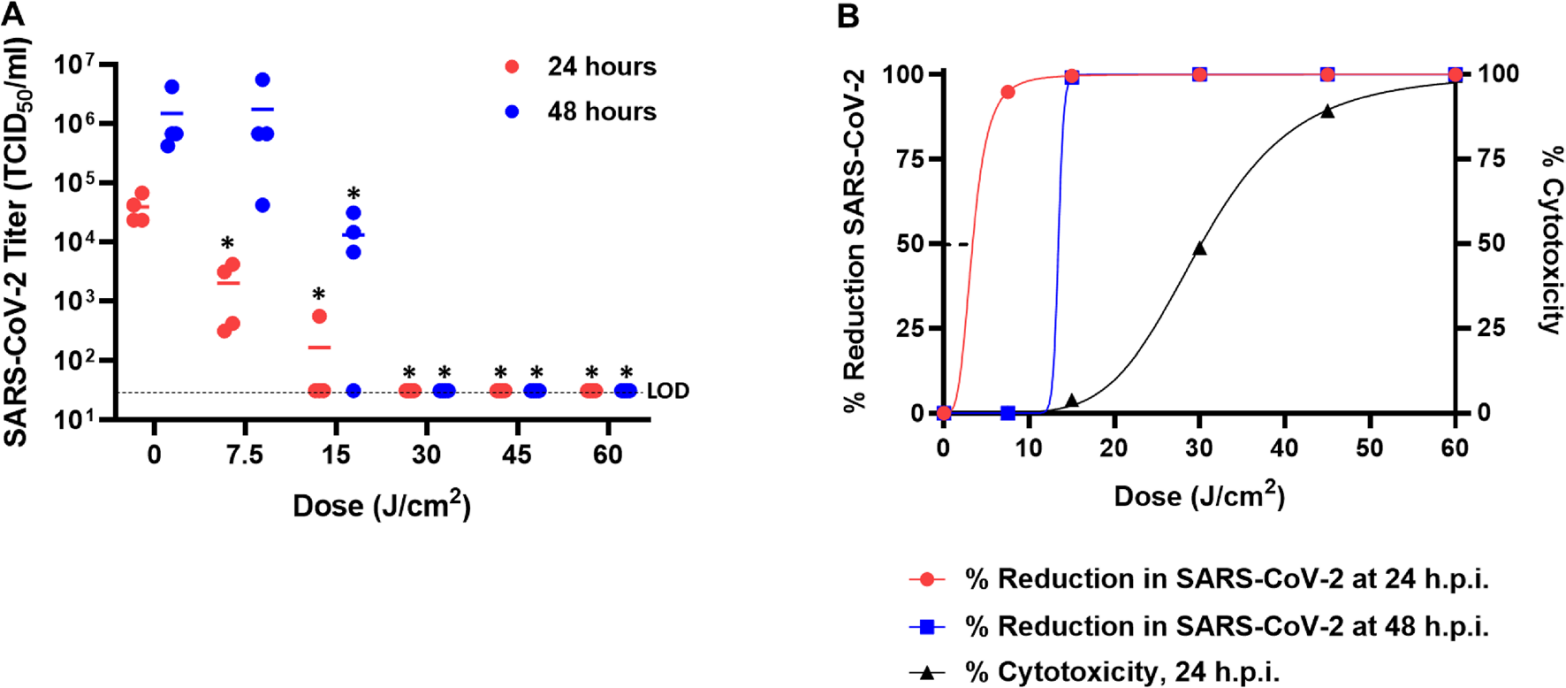
425 nm visible light inhibits cell-associated SARS-CoV-2 infection and replication on Vero E6 cells (MOI 0.01). Vero E6 cells infected with SARS-CoV-2 at a MOI of 0.01 were exposed to the indicated doses of 425 nm light at 1-h post-infection. (**A**) SARS-CoV-2 samples were harvested for TCID_50_ assays at 24- and 48-h post-infection (red and blue circles, respectively). The limit of detection (LOD) is indicated by the dashed line. (**B**) Percent reduction in SARS-CoV-2 virus was calculated at 24- and 48-h post-infection (red and blue lines, respectively). Cell viability was evaluated on Vero E6 cells that were not infected with virus and is represented as % cytotoxicity (black triangles/line). Data are represented as the mean of four independent measurements. Statistical analysis was executed using a Mann–Whitney test wherein * is *p* < 0.05 when compared to the 0 J/cm^2^ control.

The use of visible light to inactivate cell-free and cell-associated coronaviruses is unprecedented. To assess the antiviral activity of 425 nm blue light against SARS-CoV-2, Vero E6 cells were infected with a multiplicity of infection (MOI) of 0.01 SARS-CoV-2 isolate USA-WA1/2020 for 1 h. At 1-h post-infection (h.p.i.) the cell-associated virus was treated with a single illumination of 425 nm blue light at doses ranging from 7.5 to 60 J/cm^2^ (Fig. 4). At 24 h.p.i. there was a clear dose-dependent decrease in SARS-CoV-2 TCID_50_/mL (Fig. 4A). Low doses of 425 nm light were sufficient to reduce infectious SARS-CoV-2 by > 1 log with 7.5 J/cm^2^, > 2 logs with 15 J/cm^2^, and > 3 log reduction with 30 J/cm^2^. A similar trend was observed at 48 h.p.i., although continued viral replication may account for the similarity in TCID_50_/mL observed at low doses between 0 and 7.5 J/cm^2^ (Fig. 4A). These data demonstrate that 425 nm blue light interferes with SARS-CoV-2 infection in a dose-dependent manner. At doses of light that have little-to-moderate impact on the viability of Vero E6 cells (7.5, 15, and 30 J/cm^2^), we observed up to a 99.9% reduction in SARS-CoV-2 infection (Fig. 4B). Notably, cell viability was incrementally lower at 45 and 60 J/cm^2^ than the data shown in Fig. 2; however, slight variations in the cytotoxicity assay are anticipated as the SARS-CoV-2 experiments were executed in independent laboratories with differences in cell seeding density, cell passage, and cell media. Decreasing the MOI to 0.001 yielded a similar dose-dependent reduction in SARS-CoV-2 replication (Supplementary Figure 2). Despite the higher amount of input virus at MOI 0.01, a short, 2.5-min (7.5 J/cm^2^) illumination with 425 nm blue light was sufficient to reduce SARS-CoV-2 replication by > 1-log at 24 h.p.i. Consistent with these data, a similar trend in the dose-dependent effects of 425 nm blue light on SARS-CoV-2 replication was obtained by a second, independent laboratory evaluation using Vero 76 cells infected with a MOI of 0.01 at 48 h.p.i. (Supplementary Figure 3). Importantly, the dose-dependent trend showed similar log reductions despite differences in cell type (Vero 76), SARS-CoV-2 virus stock preparation, cell culture media, and viability assay. Moreover, delayed exposures of light to the Vero E6 cells at different times post infection (1, 3, 8, and 16 h.p.i.) were evaluated to explore if there were potential time points in the viral life cycle where viral replication was more sensitive to blue light. Regardless of the time of treatment, the effects of 7.5 and 15 J/cm^2^ of 425 nm light were largely consistent, with statistically significant reductions observed across all time points evaluated (Supplementary Figure 4).

An independent experimental evaluation of SARS-CoV-2 RNA showed a dose-dependent reduction in SARS-CoV-2 genomic RNA (Supplementary Tables 1 and 2); further substantiating the impact of 425 nm light on SARS-CoV-2. The fold reduction between the doses of 425 nm light with RT-qPCR detection is lower than those observed for infectious virus (TCID_50_ detection), indicating that SARS-CoV-2 viral RNA is readily detectable despite decreases in infectious virions.

### 425 nm blue light inactivation of betacoronaviruses

**Figure 5:**
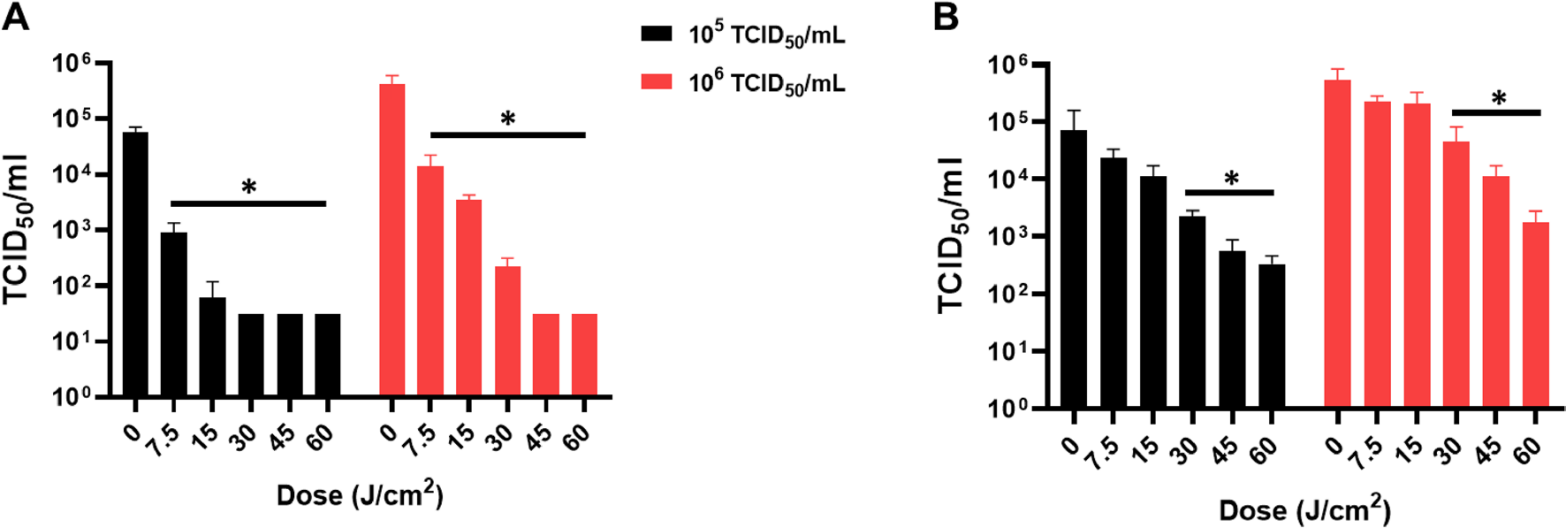
425 nm blue light exhibits dose-dependent activity against cell-free SARS-CoV-2. Two independent laboratories exposed 10^5^ and 10^6^ TCID_50_/mL SARS-CoV-2 particles to the indicated doses of 425 nm blue light. Particles exposed to light were either titered on Vero E6 cells from MRIGlobal laboratory (**A**) or Vero 76 cells from USU laboratory (**B**). Data are represented as ± SD. Statistical analysis was executed using a Mann–Whitney test wherein * is *p* < 0.05 when compared to the 0 J/cm^2^ control.

The efficacy of 425 nm blue light against cell-associated SARS-CoV-2 can result from a combination of blue light eliciting an antiviral environment in the cells and inactivating cell-free virions. To address these possibilities, cell-free SARS-CoV-2 inactivation was evaluated by two independent laboratories (Fig. 5). Two different virus suspensions containing the equivalent of ~ 10^5^ and ~ 10^6^ TCID_50_/mL SARS-CoV-2 were illuminated with the indicated doses of 425 nm blue light (Fig. 5). Following illumination, the virus was assayed by TCID_50_ on Vero E6 cells (Fig. 5A, laboratory 1) or Vero 76 (Fig. 5B, laboratory 2) cells. At laboratory 1, low doses of 425 nm light were sufficient to inactivate 10^6^ TCID_50_/mL SARS-CoV-2 > 1 log at 7.5 J/cm^2^ (> 90%), > 2 logs at 15 J/cm^2^ (> 99%), > 3 logs at 30 J/cm^2^ (> 99.9%), and > 4 logs at 60 J/cm^2^ (> 99.99%) (Fig. 5A). A similar trend in the data was observed at laboratory 2 in Vero 76 cells. Despite a less dramatic reduction in SARS-CoV-2 inactivation, a > 2 log change was still observed at 60 J/cm^2^ (> 99%) (Fig. 5B). Technical differences between laboratories including SARS-CoV-2 virus stock preparation, cell culture media, and cell types used for assaying virus may be factors that influenced the magnitude of susceptibility. Overall, the results from two independent laboratories demonstrated that low doses of 425 nm blue light (≤ 15 J/cm^2^) effectively inhibits the infection and replication of cell-free and cell-associated SARS-CoV-2 with minimal impact on cell viability.

**Figure 6:**
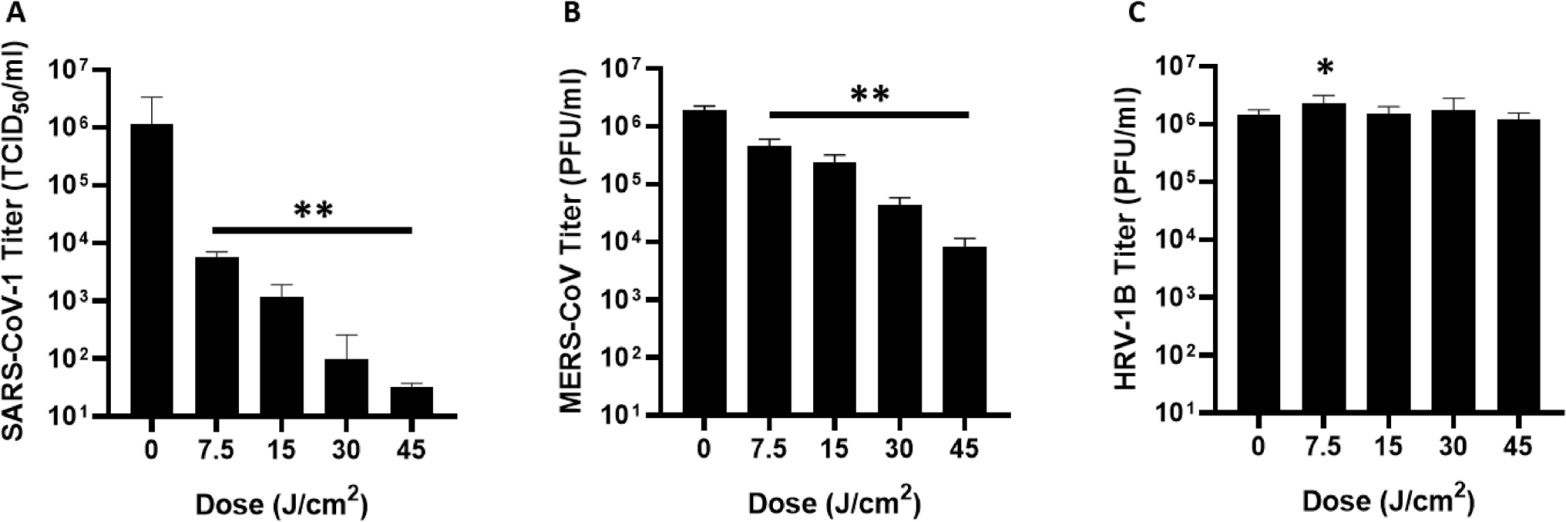
425 nm blue light exhibits dose-dependent activity against the betacoronavirus family. Independent laboratories exposed (**A**) 10^6^ TCID_50_/mL SARS-CoV-1 (MRIGlobal), (**B**) 10^6^ PFU/mL MERS-CoV (EmitBio), or (**C**) 10^6^ PFU/mL human rhinovirus 1B (EmitBio) to the indicated doses of 425 nm blue light. Particles exposed to light were titered on Vero E6 (SARS-CoV-1), Vero CCL-81 (MERS-CoV), or H1 HeLa (HRV-1B) cells. Data are represented as ± SD. Statistical analysis was executed using a Mann–Whitney test wherein * is *p* < 0.05 and ** is *p* < 0.01 when compared to the 0 J/cm^2^ control.

Expanding the cell-free data set, genetically distinct betacoronaviruses (SARS-CoV-1 and MERS-CoV) were exposed to doses of 425 nm blue light up to 45 J/cm^2^ (Fig. 6A,B). Similar to SARS-CoV-2, both SARS-CoV-1 and MERS-CoV were inactivated in a dose-dependent manner. However, MERS-CoV exhibited less inactivation with 45 J/cm^2^ than SARS-CoV-1 or SARS-CoV-2, indicating that higher doses of light may be necessary to inactivate MERS-CoV to levels observed for SARS-CoV-1 and SARS-CoV-2. Furthermore, similar doses of 425 nm light did not inactivate human rhinovirus 1B (Fig. 6C), indicating that the inactivation properties observed with betacoronaviruses may not extend to the picornavirus family and further suggests that blue light inactivates SARS-CoV-2 independently of RNA damage.

### 425 nm blue light inhibits infection/replication of SARS-CoV-2 on primary human respiratory tissue

**Figure 7:**
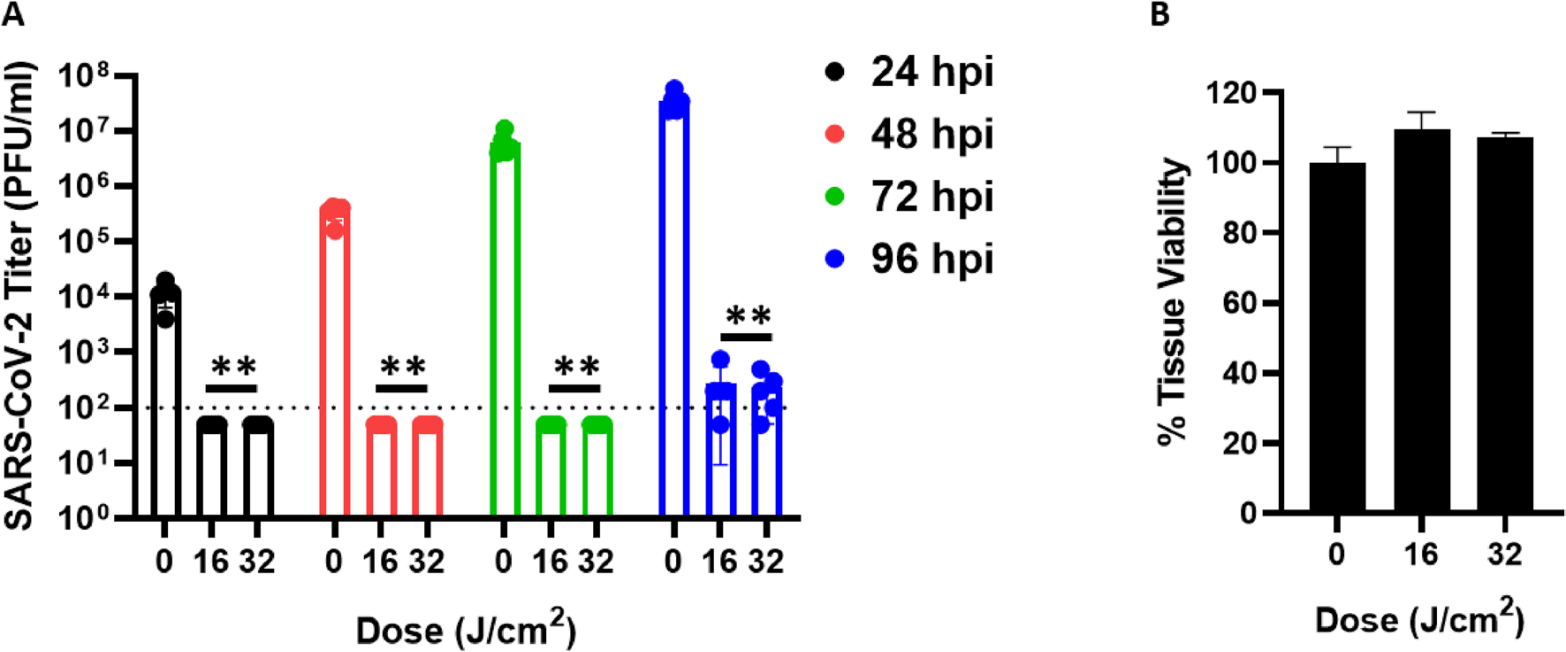
Clinically relevant doses of 425 nm blue light exhibit antiviral activity against SARS-CoV-2 in primary human airway epithelial tissue models. Primary human airway tissues were infected with SARS-CoV-2 at MOI 0.1. Infected tissues were exposed to either 16 J/cm^2^ or 32 J/cm^2^ of 425 nm light twice-daily starting at 3 h post-infection. SARS-CoV-2 samples were harvested from the apical side at 24-, 48-, 72-, and 96-h post-infection (h.p.i). (**A**) Significant reductions in SARS-CoV-2 titers were observed at all times post-infection compared to the 0 J/cm^2^ exposure control. Data are represented as a mean of five independent tissue measurements. (**B**) Tissue viability was evaluated on primary human tissues that were not infected with SARS-CoV-2. Tissue viability was evaluated after four twice-daily doses of 425 nm light at the indicated doses. Data are represented as the mean ± SD of five independent measurements. Statistical analysis was executed using a Mann–Whitney test wherein ** is *p* < 0.01 when compared to the 0 J/cm^2^ control.

The effectiveness of 425 nm blue light observed against cell-associated SARS-CoV-2 on Vero cell lines may not be the most translationally relevant cellular model when considering the development of light-based therapeutics for human applications. Primary human airway-derived 3D tissue models have significant advantages over cell based assays and have been demonstrated to support replication of SARS-CoV-2^26^. Tissue models were obtained from the Marsico Lung Institute tissue culture procurement facility at the University of North Carolina at Chapel Hill (UNC-CH) and protocols to optimize SARS-CoV-2 infection were employed to evaluate the efficacy of clinically applicable doses of 425 nm light to inhibit SARS-CoV-2 infection and replication. Repeated, twice-daily treatment with 16 or 32 J/cm^2^ doses of 425 nm light significantly inhibited infection and replication of wild-type SARS-CoV-2 at 24-, 48-, 72-, and 96-h post-infection (Fig. 7A). The dosing regimen was chosen based on an approach currently being pursued in clinical trials^27,28^. Twice daily dosing for a period of four days on the same tissue samples, in the absence of infection, demonstrated that the dosing regimen is well tolerated by human respiratory tissues after 96 h (Fig. 7B). These data indicate that a tolerable, clinically applicable regimen of 425 nm light can serve as an effective antiviral for SARS-CoV-2, and that the mechanism of action is not through photoinduced cell death.

## Discussion

The need for expedited therapeutic countermeasures against SARS-CoV-2 warrants the rapid development of novel approaches that may complement existing public health measures and FDA approved vaccines. In this study, custom BLUs were carefully engineered so that it could be demonstrated, for the first time, that safe, visible blue light can inhibit both cell-free and cell-associated SARS-CoV-2 infection and replication in a dose-dependent manner. Results from two independent laboratories demonstrate that low doses of 425 nm blue light (≤ 15 J/cm^2^) effectively inhibit infection and replication of SARS-CoV-2 (> 99%) in vitro, with minimal impact on Vero E6 cell viability. Importantly, clinically applicable regimens of 425 nm light are an effective antiviral therapy and do not elicit cytotoxicity in 3D tissue models established from human large airway epithelial cells.

The 3D tissue models established from large human airways are in vitro organotypic models of human mucociliary airway epithelium cultured at the air/liquid interface to provide a differentiated in vivo-like epithelial structure with barrier properties and metabolic functions^29^. There is strong global momentum to replace animal model testing with relevant in vitro human-derived test systems to reduce the number of animals used in preclinical testing. Current testing guidelines (TG403, TG433, and TG436), established by the Organization for Economic Co-operation and Development (OECD), for inhalation toxicity outline the use of animals to determine the LC_50_ (concentration required to cause death of 50% of the test animals) of therapeutics. The commercially available EpiAirway in vitro tissue model can be used to determine the IC_25_ value (concentration required to reduce tissue viability by 25% of vehicle control-treated tissues) of a test article^29^. Following 3-h of exposure to the test article, the model has been shown to predict respiratory tissue viability using chemicals with the Globally Harmonized System (GHS) Acute Inhalation Toxicity Category 1 and 2, and Environmental Protection Agency (EPA) Acute Inhalation Toxicity Category I-II classifications^29^. Extended exposure times (24 and 72 h) with toxic chemicals also reflect in vivo responses, demonstrating the predictive value of the EpiAirway models for respiratory toxins in humans^30^. Furthermore, such a uniform in vitro model is ideally suited to evaluate the safe doses of light applied to a fixed surface area (J/cm^2^) rather than attempting to scale the optical delivery of light to the appropriate animal model anatomy.

In this study the EpiAirway model was exposed to a dose range of 385 nm, 405 nm, and 425 nm light. Exposure to UVA light at 385 nm exhibited > 25% loss in viability at ≥ 45 J/cm^2^, identifying a dose that breaches the IC_25_ threshold established for acute cytotoxicity in the EpiAirway model. In contrast, higher doses of the 425 nm blue light did not reach the IC_25_ threshold for validated acute airway irritation. Greater than 100% tissue viability was observed following illumination with antiviral (> 99.99% reduction in SARS-CoV-2) 425 nm blue light doses of 60 J/cm^2^. The distinct viability profiles observed at 385 nm, 405 nm, and 425 nm demonstrate that the 3D EpiAirway tissue models are amenable for identifying acute respiratory effects associated with light therapy in a dose- and wavelength-dependent manner. Minimal losses in viability when doses as high as 120 J/cm^2^ are applied at 425 nm indicates that the 3D human respiratory tissue models are highly tolerant to this wavelength. This was further substantiated by the fact that twice daily dosing of 3D human respiratory tissue with 32 J/cm^2^ was well tolerated following receipt of a total of 256 J/cm^2^ after 4 days (MLI model).

The 2D Vero E6 cell cultures exhibited a cell density-dependent viability response to 425 nm doses ≥ 15 J/cm^2^, wherein low seeding densities per surface area were more susceptible to light-induced cytotoxic effects. The enhanced tolerance of the 3D human respiratory tissue models to 425 nm blue light compared to 2D Vero E6 cell cultures is not surprising, given that cells in 3D culture are often more resistant to drug treatment, drug metabolism is more effective, and there is increased resistance to drug-induced apoptosis^31^. The characteristics of 3D tissue models more closely reflect cellular attributes observed in the context of tissues in vivo. Developing optimal conditions for SARS-CoV-2 infection and replication in 3D respiratory tissue models, including other anatomical regions of the respiratory tract (e.g. human oral, nasal, and lower respiratory tract), will help to further elucidate mechanisms that govern the ability of visible light to inactivate SARS-CoV-2.

The molecular mechanisms governing the impact of blue light on non-pigmented cells are only beginning to be revealed. The effects of blue light should follow the first law of photochemistry which states that light must be absorbed to have an effect. A handful of photoacceptors for blue light have been identified in non-pigmented cells, including cytochrome c oxidase, flavins, porphyrins, opsins, and nitrosated proteins^32,33^. Light absorption by photoreceptors can lead to, for example, the release of reactive oxygen species (ROS) and/or nitric oxide (NO) that may function to inactivate SARS-CoV-2 in a cell-free or cell-associated environment. Reactive oxygen species and/or bioactive NO might elicit activation of transcription factors involved in immune signaling, such as nuclear factor kappa-light-chain-enhancer of activated B cells (NFκB) and mitogen activated protein kinase (MAPK) signaling^32^. NFκB and MAPK pathways can lead to transcriptional activation of innate and inflammatory immune response molecules that may interfere with SARS-CoV-2 replication^32,34,35^. Nitric oxide can also mediate inactivation of cell-associated SARS-CoV-2 through S-nitrosation of cysteine residues in the active site of viral encoded enzymatic proteins. Evidence demonstrating that SARS-CoV-1 and SARS-CoV-2 can be inactivated by exogenous addition of NO donor molecules substantiate the potential for SARS-CoV-2 inactivation by nitric oxide^36–38^. Photosensitizers present in cell media may additionally facilitate generation of ROS and/or NO that directly impact virion proteins and/or viral RNA to prevent infection and replication^20^.

Studies in this manuscript are the first to demonstrate the utilization of safe, visible blue light to inhibit SARS-CoV-2 replication in infected human tissue without damage to healthy cells/tissue. Prior UV studies demonstrated the inactivation of SARS-CoV-2, and other coronaviruses, only under cell-free conditions, and only with light in the UVC range (200–280 nm)^12–14^. For example, ultraviolet light, especially the use of UVC lamps, is being widely considered for irradiating surfaces that may harbor SARS-CoV-2. In response, the FDA issued a statement on its website cautioning against the personal use of these devices for inactivating SARS-CoV-2^39^. In the statement, the FDA warns that “UVC radiation can cause severe burns (of the skin)” and recommends to “avoid direct skin exposure to UVC radiation.” Importantly, UV radiation is highly mutagenic through induced formation of pyrimidine dimers in genomes resulting in genomic instability, which can ultimately lead to cancer^15,16^. Considering these risks, widespread clinical human applications of UV light should be avoided without significant additional human trials.

The studies presented here demonstrated that 425 nm blue light can achieve inactivation of genetically distinct betacoronaviruses including SARS-CoV-1, SARS-CoV-2, and MERS-CoV in the absence of UV light. When compared to the inability of 425 nm to inactivate HRV-1B, these data indicate that structural attributes of coronaviruses may have a unique susceptibility to inactivation by low doses of 425 nm blue light and that the mechanism is unlikely to be related to non-specific, photo-oxidation of nucleic acids. Furthermore, unlike studies with UV light, cell-associated studies with 425 nm blue light exhibited minimal cytotoxicity at doses effective at reducing SARS-CoV-2 infection and replication. This was especially pronounced in the human 3D human respiratory tissue models in which the tissues tolerated high doses of 425 nm blue light (up to 120 J/cm^2^ for EpiAirway model and 256 J/cm^2^ through 96 h for the MLI model). The effective use of blue light presented in these this studies suggest that the safe use of 425 nm blue light should be considered for clinical human applications, albeit, under the auspices of FDA guidance.

Several studies demonstrate high SARS-CoV-2 viral loads in the upper respiratory tract of individuals, especially in the nasopharyngeal and oropharyngeal cavities^40–44^. Higher viral loads may lead to more severe symptoms associated with COVID-19 and a higher risk of transmissibility^40,42^, however, there are no therapeutics approved for use that may reduce SARS-CoV-2 viral load locally in the upper respiratory tract. Importantly, clinically relevant doses (≤ 32 J/cm^2^) of 425 nm light inhibit SARS-CoV-2 infection and replication in primary human 3D tissue models and exhibited minimal impact on tissue viability across two independent models. Applying low doses of 425 nm blue light to the upper respiratory tract may reduce high viral loads in the upper respiratory tract, which may in turn curb human-to-human transmission, as well as subsequent infection of the lower respiratory tract. In vitro studies reported here demonstrate the feasibility of using 425 nm blue light to interfere with the infection and replication of SARS-CoV-2 through a mechanism that does not result in cell death. Although these studies are far removed from a clinical application, ongoing clinical trials are currently evaluating the use of safe, visible blue light as a treatment for COVID-19^27,28^.

## Materials and methods

### Cells, tissues, and viruses

At EmitBio, Vero E6 and Vero CCL-81 cells were purchased from ATCC and maintained in DMEM (Sigma-Aldrich) supplemented with 10% FetalCloneII (HyClone) and 1% Antibiotic–Antimycotic (Gibco). The H1 HeLa cells were procured from ATCC (CRL-1958) and maintained in DMEM (Sigma) supplemented with 10% FetalCloneII serum (HyClone) and 1% antibiotic–antimycotic (Gibco). At MRIGlobal, Vero E6 cells were propagated and maintained in DMEM/F12 (Gibco) supplemented with 10% FBS (Avantor). At Utah State University, Vero 76 cells (ATCC CRL-1587) were maintained in MEM supplemented with 2 mM l-glutamine and 5% FBS.

Primary human tissue models, derived from large airway epithelial cells, were acquired from two different suppliers. The first supplier provided primary human airway epithelium (EpiAirway AIR-100, MatTek Corporation) cultured for 28 days in transwell inserts by MatTek Corporation. The cultured tissues were shipped in 24-well plates with agarose embedded in the basal compartment. Upon arrival, the transwell inserts were removed and placed in 6-well plates with a proprietary, cold maintenance media in the basal compartment; no media added to the apical surface. Tissues were incubated at 37 °C and 5% CO_2_ overnight prior to experimental use. The second supplier provided differentiated primary human tissue models (derived from donor DD065P, a 27 year old female) grown and differentiated at the Marsico Lung Institute (MLI) Tissue Procurement and Cell Culture Core facility at the University of North Carolina at Chapel Hill, as previously described^45^. Briefly, day 28 cultures were prepared in basolateral ALI media for 3–5 days prior to experimental initiation with media change every other day. For SARS-CoV-2 infections of these cultures, SARS-CoV-2 WA1/2020 P4 was diluted in virus diluent [MEM supplemented with 2% FBS (Gibco), 1% nonessential amino acids (Gibco), and 1% antibiotic–antimycotic (Gibco)]. Transwell cultures were inoculated with 200 µL of diluted virus (MOI 0.1) for 2 h on the apical surface at 37 °C and 5% CO_2_. Following incubation, virus was removed from the apical surface and the transwell cultures were incubated at 37 °C and 5% CO_2_. Infected transwell cultures were illuminated with 0 J/cm^2^, 16 J/cm^2^, or 32 J/cm^2^ using the BLU within a biosafety cabinet two times daily. The first dose was administered 3 h after initial virus infection. Viral titers were measured at the indicated timepoints (24, 48, 72, and 96 h post-infection) via apical washes. Briefly, 200 µL of virus diluent (as above) was added to the apical surface of the transwell insert and the cultures were incubated at 37 °C and 5% CO_2_ for 30 min. After incubation, the apical wash was removed and centrifuged at 5000 rpm for 5 min and stored at − 80 °C. Viral titers were determined via plaque assay (described below).

All work with live virus was conducted in three independent Biosafety Level-3 (BSL-3) laboratories, MRIGlobal’s Kansas City facility (laboratory 1), the Institute for Antiviral Research at Utah State University (laboratory 2), and the newly established, CDC certified, BSL-3 laboratory at EmitBio, with adherence to established safety guidelines. At the MRIGlobal and Utah State University laboratories, SARS-CoV-2 (USA_WA1/2020) was obtained from the World Reference Center for Emerging Viruses and Arboviruses (WRCEVA) and propagated with slight modifications. At MRIGlobal, Vero E6 cells were cultured overnight with DMEM (Gibco; 12320-032) supplemented with 5% FBS (Avantor, 97068-085), 1% nonessential amino acids (Corning 25-025-Cl), and 1% penicillin/streptomycin (VWR 97063-708). To generate master stocks, cells were infected with an approximate MOI of 0.08 in infection media (as above with 5% FBS). Cells were monitored for cytopathic effect (CPE) daily and harvested at 4 days post-infection as CPE approached 100%. Working stocks were cultured in Vero E6 cells with DMEM/F12 media (Gibco; 11330-032) supplemented with 10% FBS and 1% penicillin/streptomycin at a MOI of 0.005. Cells were monitored for CPE and harvested two days post-infection as CPE approached 70%. Cell culture debris was pelleted by centrifugation at 500×*g* for 5 min and viral stocks were stored at − 80 °C. Infectivity of viral stocks was determined by TCID_50_ assay. Also at MRIGlobal, SARS-CoV-1 was cultured similar to SARS-CoV-2 except the virus was incubated for 5 days prior to harvest. SARS-CoV-1 was titered by TCID_50_ assay as described below for SARS-CoV-2. The SARS-CoV-1 (Urbani strain) virus was originally sourced from BEI Resources. All work at MRIGlobal was performed in a BSL3 laboratory certified to work with select agents.

At Utah State University, SARS-CoV-2 (USA_WA1/2020) was propagated in Vero 76 cells. Infection media was Minimal Essential Media supplemented with 2 mM l-glutamine, 2% FBS, and 50 µg/mL gentamicin.

At EmitBio, the following reagents were obtained through BEI Resources, NIAID, NIH: SARS-related coronavirus 2 isolate USA-WA1/2020 (cat # NR-52281) and Middle East Respiratory Syndrome Coronavirus (MERS-CoV), EMC/2012 (cat# NR-44260). SARS-CoV-2 isolate WA1/2020 was propagated in Vero E6 cells at an MOI of 0.001 (P1) in MEM supplemented with 5% FBS (Gibco), 1% nonessential amino acids, and 1% antibiotic–antimycotic (Gibco). Cytopathic effect was observed daily and the P1 was harvested at day 4 post-infection. The P1 stock was serially passaged on Vero E6 cells to a P4 working stock at an MOI of ~ 0.001–0.005, as determined from the number of cells seeded on the flask. The P4 stock was used in subsequent studies. The MERS-CoV P1 working stock was propagated on Vero CCL-81 cells at 37 °C and 5% CO_2_ at MOI 0.001. Human rhinovirus 1B (HRV-1B) (ATCC cat# VR-1645) was passaged twice on H1-HeLa cells at 34 °C and 5% CO_2_ at MOI 0.01 (P1) and MOI 0.0001 (P2 working stock). The rhinovirus 1B P2 stock was utilized for these studies.

### LED array calibration

After optimization of the LED Array, each system was independently calibrated to a targeted irradiance (385 nm, 405 nm, and 425 nm LED arrays). To do this, the target surface under each array, which exceeded the area of a standard multi-well plate, was divided into 12 zones in a three-row by four-column configuration. Subsequently a calibrated ILT-1400 Photometer with an SEL033 Broadband Detector (International Light Technologies, Peabody, MA) was positioned 90 mm away from the LED Array, and the irradiance was measured in each of the twelve zones with the array operating at the specified input current and a microplate cover positioned between the LED array and the detector. The measured irradiance of each of the 12 zones was averaged and verified to be within one-two percent (1–2%) of the target irradiance. The input current was adjusted, and the procedure repeated if the average irradiance of the 12 zones exceeded the limits. Lifetime studies verified that the irradiance measured at the specified current did not change significantly over the course of a single illumination or over the duration of the experiments.

The irradiance settings used in these studies for each of the 385, 405, and 425 nm arrays were 25, 50, and 50 mW/cm^2^, respectively. For example, exposure times for 425 nm light were varied to deliver different optical doses in J/cm^2^ according to the following schedule: 7.5 J/cm^2^ (150 s), 15 J/cm^2^ (300 s) 30 J/cm^2^ (600 s), 45 J/cm^2^ (900 s), 60 J/cm^2^ (1200 s), 90 J/cm^2^ (1800 s), 120 J/cm^2^ (2400 s).

The spectral flux of each LED Array was measured in a 1.5 m Integrating Sphere Spectroradiometer with CD610 Spectrometer (Labsphere, North Sutton, NH) using the same input current required to deliver the targeted irradiance. The peak wavelength and spectral flux for each LED array was determined and verified using the associated Labsphere Integral Software.

### Cytotoxicity assays for human tissues

The EpiAirway tissues are supplied as a kit (AIR-100: MatTek Corporation, Ashland, MA) and were prepared following the supplier’s recommendations. Prior to illumination, the maintenance media (AIR-100-MM) was changed on the human tissue transwell inserts. Tissues were illuminated at room temperature with 385 nm, 405 nm, or 425 nm light and incubated at 37 °C and 5% CO_2_ for 3 h. Cytotoxicity was assayed using 3-(4,5-dimethylthiazol-2-yl)-2,5-diphenyltetrazolium bromide (MTT) following manufacturer’s instructions (MTT-100, MatTek corporation). Briefly, tissues were rinsed with TEER buffer and placed into 300 µL of pre-warmed MTT reagent and incubated at 37 °C and 5% CO_2_ for 90 min. The MTT stained tissue inserts were then extracted with 2 mL of MTT extractant solution by shaking at 85 rpm for 2 h on an orbital shaker. The tissue inserts were discarded and 200 µL of the extractant solution was added to a clear 96-well plate, in duplicate, and absorbance was measured at 560 nm on a GloMax Discover (Promega). Extractant solution served as the experimental blank and cell viabilities were calculated against plates that were not illuminated. The viability of light-exposed tissues was calculated relative to dark controls using the equation: Relative viability = [OD_sample_/Mean OD_neg ctrl_] × 100. All control and treatment groups included five replicates. Moreover, the MTT assay described here was adapted to assess cytotoxicity of light on human tissue models procured from the Marsico Lung Institute Tissue Procurement and Cell Culture Core facility at UNC-CH.

### Cytotoxicity assays for cell lines

At EmitBio and MRIGlobal Vero E6 cells were incubated in clear 24-well, 48-well, and 96-well plates (Corning) at varying seeding densities and incubated at 37 °C and 5% CO_2_ overnight. Cells were illuminated with 425 nm light and incubated at 37 °C and 5% CO_2_ for 24 h post-illumination. After 24 h, cytotoxicity was determined using the CellTiterGlo One Solution (Promega) with modifications. The amount of CellTiterGlo One Solution was optimized in a preliminary experiment. For 24-well plates, 100 µL solution was used and 60 µL solution was used for 48- and 96-well plates. The cells were placed on an orbital shaker for 2 min and the chemiluminescent signal was stabilized for 10 min before 50 µL of the solution was added to a black well, black bottom 96-well plate and read using the CellTiterGlo program on the GloMax (Promega). CellTiterGlo One solution served as a blank and cell viabilities were calculated against plates that were not illuminated.

At Utah State University, cytotoxicity analysis was conducted at 48 h post-illumination. Cells were treated for 2 h with 0.01% neutral red for cytotoxicity. Excess dye was rinsed from cells with PBS. Absorbed dye was eluted from the cells with 50% Sorensen’s citrate buffer/50% ethanol for 30 min. Buffer was added to 10 wells per replicate. Optical density was measured at 560 nm. Cell viabilities were calculated against cells that were not illuminated.

### Cell-associated virus assays

Cell-associated virus assays were conducted in separate laboratories with modifications. At MRIGlobal, cells were infected with SARS-CoV-2 at approximate multiplicity of infections (MOI) of 0.01 and 0.001. These values were based on the number of cells seeded in a single well. For each experiment Vero E6 cells were seeded at 1.5 × 10^5^ cells/well in a 24-well plate format. At one-hour post-infection, infected cells were illuminated with 425 nm light at the specified doses. Cell culture supernatants were harvested at 24 h and 48 h post-infection to for TCID_50_ determination and qPCR analysis. No illumination controls and no virus controls were included as a positive control for viral growth and for cytotoxicity, respectively. Cytotoxicity analysis were conducted at 24 h post-illumination as above.

At Utah State University, Vero 76 cells were infected with SARS-CoV-2 at approximate MOIs of 0.01 and 0.001. These values were based on the number of cells seeded in a single well. For each experiment Vero 76 cells were seeded at 2 × 10^5^ cells/well in a 24-well plate format. At one-hour post-infection, infected cells were illuminated with 425 nm light at the specified doses. Cell culture supernatants were harvested at 48 h post-infection for TCID_50_ determination. No illumination controls and no virus controls served as a positive control for viral growth and for cytotoxicity, respectively. Cytotoxicity analysis were conducted at 48 h post-illumination.

### Cell-free virus assays

SARS-CoV-2 cell-free virus assays were conducted in parallel in separate laboratories. At MRIGlobal, 1 mL solutions containing 10^5^ and 10^6^ TCID_50_/mL of SARS-CoV-2 or SARS-CoV-1 were illuminated with varying doses of light. The viruses were then titered on Vero E6 cells in triplicate via TCID_50_ assay. No illumination controls served as a positive control for viral growth.

At Utah State University, 1 mL solutions containing 10^5^ and 10^6^ TCID_50_/mL of SARS-CoV-2 were illuminated with varying doses of light. The viruses were then titered on Vero 76 cells in triplicate via TCID_50_ assay. No illumination controls served as a positive control for viral growth.

MERS-CoV and human rhinovirus 1B assays were conducted at EmitBio. For MERS-CoV, 500 µL containing 10^6^ p.f.u. of MERS-CoV P1 working stock in MERS-CoV virus diluent [high glucose DMEM (Sigma) supplemented with 2.5% FetalClone II (HyClone) and 1% antibiotic–antimycotic] were illuminated with the indicated doses of 425 nm light. Viruses were titered via plaque assay on Vero CCL-81 cells as described below. For HRV-1B, 500 µL containing 10^6^ p.f.u. of P2 working stock in rhinovirus diluent [high glucose DMEM (Sigma) supplemented with 2.5% FetalClone II (HyClone) and 1% antibiotic–antimycotic] were illuminated with the indicated doses of 425 nm light. Viruses were titered via plaque assay on H1 HeLa cells.

### RT-qPCR

All RNA work was conducted at MRIGlobal. Viral RNA levels for SARS-CoV-2 samples were determined by quantitative RT-PCR using the CDC N1 assay. Samples for the RT-PCR reactions were live virus in culture supernatants without nucleic acid extraction. Primers and probes for the N1 nucleocapsid gene target region was sourced from Integrated DNA Technologies (2019-nCoV CDC RUO Kit, No. 10006713). TaqPath™ 1-step RT-qPCR Master Mix, CG was sourced from ThermoFisher (No. A15299). Reaction volumes and thermal cycling parameters followed those published in the CDC 2019-Novel Coronavirus (2019-nCoV) Real-Time RT-PCR Diagnostic Panel: Instructions for Use. For the RT-PCR reaction, 15 µL of prepared master mix was added to each well followed by 5 µL of each sample, for a final total volume of 20 µL per reaction well. Reactions were run on a Bio-rad CFX real-time PCR instrument.

### Tissue culture infectious dose 50 (TCID_50_) determination

TCID_50_ assays were conducted as follows at both MRIGlobal and Utah State University with slight modifications. At MRIGlobal, Vero E6 cells were plated in 96-well plates at 10,000 cells/well in 0.1 mL/well of complete medium (DMEM/F12 with 10% fetal bovine serum and 1 × Penicillin/Streptomycin) and incubated overnight in a 37 °C, 5% CO_2_ humidified incubator. The next day virus samples were serially diluted into unsupplemented DMEM/F12 media at 1:10 dilutions by adding 0.1 mL virus to 0.9 mL diluent, vortexing briefly and repeating until the desired number of dilutions was achieved. Media was decanted from 96-well plates and 0.1 mL of each virus dilution aliquoted into 5 or 8 wells. After 4 days of incubation at 37 °C, 5% CO_2,_ plates were scored for presence of cytopathic effect. TCID_50_/mL were made using the Reed and Muench method^46,47^. At Utah State University, cell culture samples were serially diluted and plated on fresh Vero 76 cells in quadruplicate. Plates were visually examined for CPE at 6 days post-infection. Wells were indicated as positive or negative and virus titers were calculated using the Reed-Muench endpoint dilution method.

### Viral plaque assays

Infectious viral titers were enumerated via plaque assay in all experiments conducted at EmitBio. For SARS-CoV-2, Vero E6 cells were plated in 12-well tissue-culture treated plates at ~ 1.5 × 10^5^ cells/well and incubated overnight at 37 °C and 5% CO_2_. Samples were serially diluted in SARS-CoV-2 diluent (described above) from 10^–1^ to 10^–6^. Vero E6 cells were inoculated with 100 µL of each diluted sample for 1 h with gentle rocking every 15 min at 37 °C and 5% CO_2_. After 1 h, 1 mL of 0.6% carboxymethylcellulose (CMC) (Sigma) overlay [MEM supplemented with 2% FBS (Gibco), 1% sodium pyruvate (Gibco), 1% nonessential amino acids (Gibco), 0.3% sodium bicarbonate (Gibco), 1% GlutaMAX (Gibco), and 1% antibiotic–antimycotic (Gibco)]. Plaque assays were fixed with 1 mL 10% neutral-buffered formalin and stained with 0.125% crystal violet.

To execute plaque assays for MERS-CoV, Vero CCL-81 cells were plated in 12-well tissue culture plates at ~ 1.5 × 10^5^ cells/well and incubated overnight at 37 °C and 5% CO_2_. Samples were serially diluted in MERS-CoV virus diluent (described above) from 10^–1^ to 10^–6^. Vero CCL-81 were inoculated with 100 µL of each diluted sample for 1 h with gentle rocking every 15 min at 37 °C and 5% CO_2_. After 1 h, 1 mL of 1.2% colloidal microcrystalline cellulose (Sigma) overlay [high glucose DMEM supplemented with 2.5% FetalClone II (HyClone), 1% sodium pyruvate (Gibco), 1% nonessential amino acids (Gibco), 5% sodium bicarbonate (Gibco) and 1% Antibiotic–antimycotic (Gibco)] was added. Plaque assays were fixed with 1 mL 10% neutral-buffered formalin and stained with 0.125% crystal violet.

For HRV-1B, H1 HeLa cells were plated in 12-well tissue culture treated plates at ~ 2.5 × 10^5^ cells/well and incubated overnight at 37 °C and 5% CO_2_. Samples were serially diluted in HRV-1B diluent (described above) from 10^–1^ to 10^–6^. H1 HeLa cells were inoculated with 100 µL of each diluted sample for 1 h with gentle rocking every 15 min at 34 °C and 5% CO_2_. After 1 h, 1 mL of 1.2% colloidal microcrystalline cellulose (Sigma) overlay [high glucose DMEM supplemented with 2.5% FetalClone II (HyClone), 1% sodium pyruvate (Gibco), 1% nonessential amino acids (Gibco), 5% sodium bicarbonate (Gibco) and 1% Antibiotic–antimycotic (Gibco)]. Plaque assays were incubated for 3 days at 34 °C and 5% CO_2_. Plaque assays were fixed with 1 mL 10% neutral-buffered formalin and stained with 0.125% crystal violet.

The limit of detection for these assays was 100 p.f.u./mL. For statistical purposes, and data representation, all samples that were under the limit of detection were set to half the limit of detection.

### Statistical analysis

Statistical significance in viral titers were determined via the Mann–Whitney ranked sum test using GraphPad Prism 8. Statistical significance is indicated by *p < 0.05 and **p < 0.01, where applicable.

## Supplementary Information

Supplementary Information.
