# Supplementary material for: Visible blue light inhibits infection and replication of SARS-CoV-2 at doses that are well-tolerated by human respiratory tissue
Source: Sci Rep. 2021 Oct 18;11:20595. doi: 10.1038/s41598-021-99917-2 (PMC8523529; doi:10.1038/s41598-021-99917-2)
Supplement: Supplementary file 1 — Supplementary Information. [file 41598_2021_99917_MOESM1_ESM.pdf]

Supplementary Information

Visible blue light inhibits infection and replication of SARS-CoV-2 at doses that are well-tolerated by human respiratory tissue

Authors: Nate Stasko[a], Jacob F. Kocher[a], Abigail Annas[a], Ibrahim Henson[a], Theresa S. Seitz[b], Joy M. Miller[b], Leslee Arwood[a], Rachel C. Roberts[a], Thomas M. Womble[a], Emily G. Keller[a], Soren Emerson[a], Michael Bergmann[a], Ashley N. Y. Sheesley[c], Rebecca J. Strong[c], Brett L. Hurst[c], David Emerson[a], E. Bart Tarbet[c], Shelton S. Bradrick[b], and Adam S. Cockrell[a,#]

Address Correspondence to A.S.C. (acockrell@knowbiollc.com)

Supplementary Tables 1-2

Supplementary Figures 1-4

Supplementary Table 1. Light dose-dependent reduction in SARS-CoV-2 RNA at MOI 0.001

Dose (J/cm^2)	24 hours post-infection				48 hours post-infection			
	Average Cq value	Standard Deviation of Cq	[b]Fold Decrease	[c]% Reduction	Average Cq value	Standard Deviation of Cq	[b]Fold Decrease	[c]% Reduction
0	22.28	5.28	1.00	--	15.11	0.23	1.00	--
7.5	21.21	0.20	0.48	[a] 0.00	14.25	0.13	0.55	[a] 0.00
15	26.95	0.38	25.52	96.08	17.18	0.14	4.21	76.24
30	28.29	0.38	64.45	98.45	24.55	1.10	696.19	99.86
45	28.37	0.18	67.96	98.53	25.21	0.24	1094.96	99.91
60	26.29	3.97	16.11	93.79	25.43	0.19	1275.34	99.92

[a]Values less than 0 are represented as 0%

[b]Fold decrease relative to 0 J/cm^2

[c]Percent SARS-CoV-2 reduction relative to 0 J/cm^2

Supplementary Table 2. Light dose-dependent reduction in SARS-CoV-2 RNA at MOI 0.01

Dose (J/cm^2)	24 hours post-infection				48 hours post-infection			
	Average Cq value	Standard Deviation of Cq	[b]Fold Decrease	[c]% Reduction	Average Cq value	Standard Deviation of Cq	[b]Fold Decrease	[c] % Reduction
0	18.35	0.90	1.00	--	13.58	0.48	1.00	--
7.5	17.99	0.34	0.78	[a] 0.00	13.65	0.32	1.05	4.74
15	21.57	2.32	9.36	89.32	15.50	0.09	3.78	73.51
30	24.92	1.06	95.01	98.95	18.54	0.86	30.98	96.77
45	25.59	0.46	151.52	99.34	22.01	0.03	343.30	99.71
60	25.33	0.78	126.82	99.21	22.03	0.10	349.71	99.71

[a]Values less than 0 are represented as 0%

[b]Fold decrease relative to 0 J/cm^2

[c]Percent SARS-CoV-2 reduction relative to 0 J/cm^2

Supplementary Figure 1

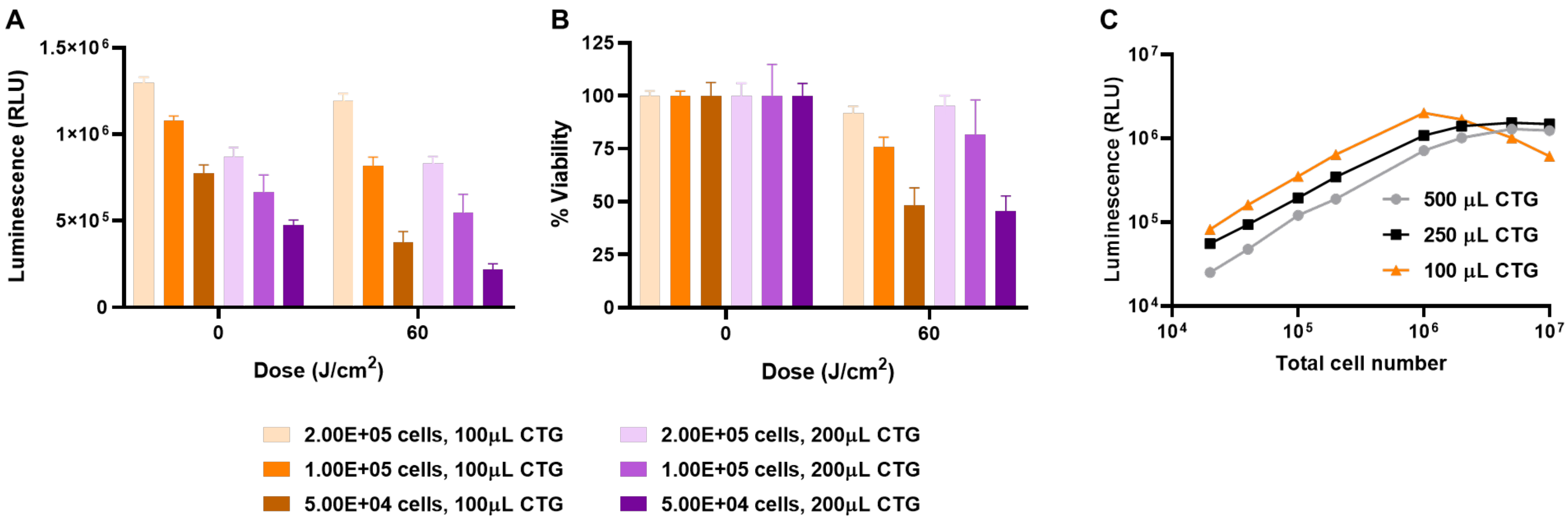

Supplementary Figure 1. Vero E6 viability as a function of CellTiter-Glo (CTG) reagent and cell density. Vero E6 cells were seeded at different seeding densities in 24 well plates and incubated overnight. Cells were illuminated with 60 J/cm^2 425 nm light. Cell viability was determined with 100 µL (orange) or 200 µL (purple) (CTG) One Solution at 24 hours post-illumination against 0 J/cm^2 controls. Data presented are the mean raw luminescence values (a) and the mean percent cell viability relative to the 0 J/cm^2 dose (b) ± SEM. (c) Specific numbers of Vero E6 cells were treated with 100 µL (orange), 250 µL (black), or 500 µL (gray) CTG One Solution. Data are represented as raw luminescence values.

Supplementary Figure 2

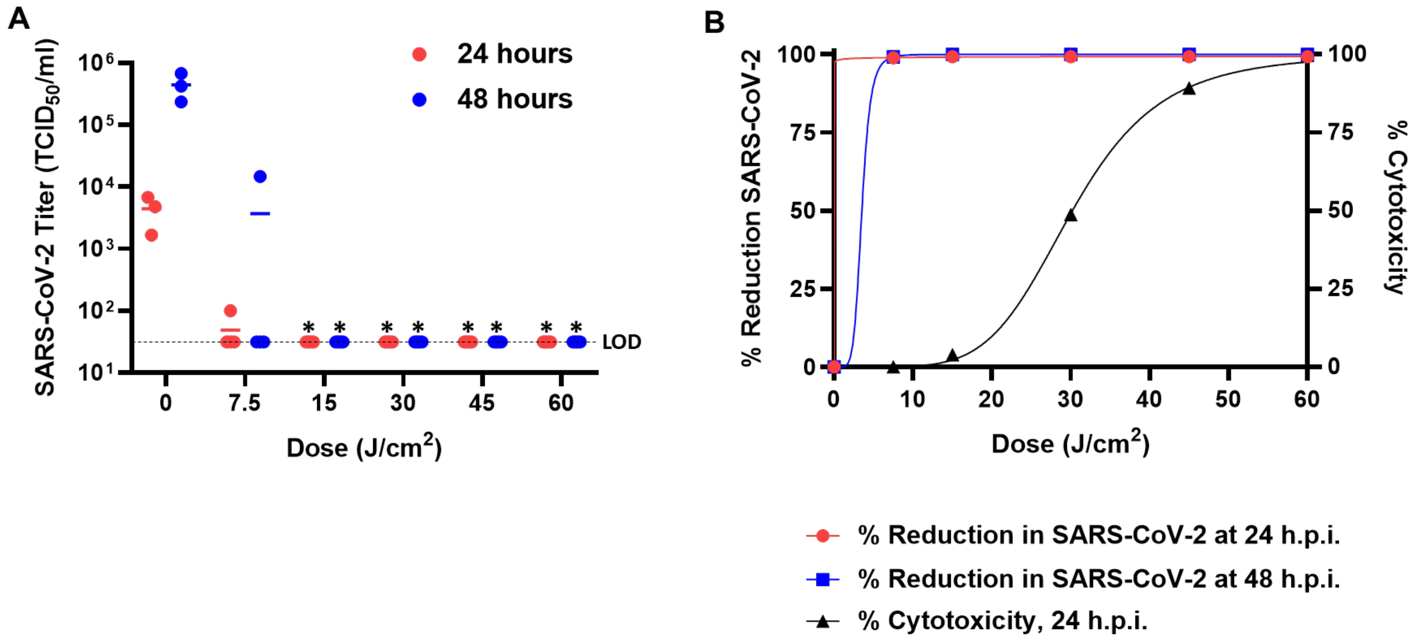

Supplementary Figure 2. 425 nm visible light inhibits infection and replication of cell-associated SARS-CoV-2 on Vero E6 cells (MOI 0.001). Vero E6 cells were infected with SARS-CoV-2 at a MOI of 0.001 and exposed to the indicated doses of 425 nm light at 1-hour post-infection. (a) SARS-CoV-2 samples were harvested for $TCID_{50}$ assays at 24- and 48-hours post-infection (red and blue circles, respectively). The limit of detection (LOD) is indicated by the dashed line. (b) Cell viability was evaluated on Vero E6 that were not infected with virus and is represented as percent cytotoxicity (black triangles/line). The percent reduction in SARS-CoV-2 virus was calculated at 24- and 48-hours post-infection (red and blue lines, respectively). Data are represented as the mean of four independent measurements except for the 0 J/cm^2, which had three independent measurements. Statistical analysis was executed using a Mann-Whitney test wherein * is $p < 0.05$ when compared to the 0 J/cm^2 control.

Supplementary Figure 3

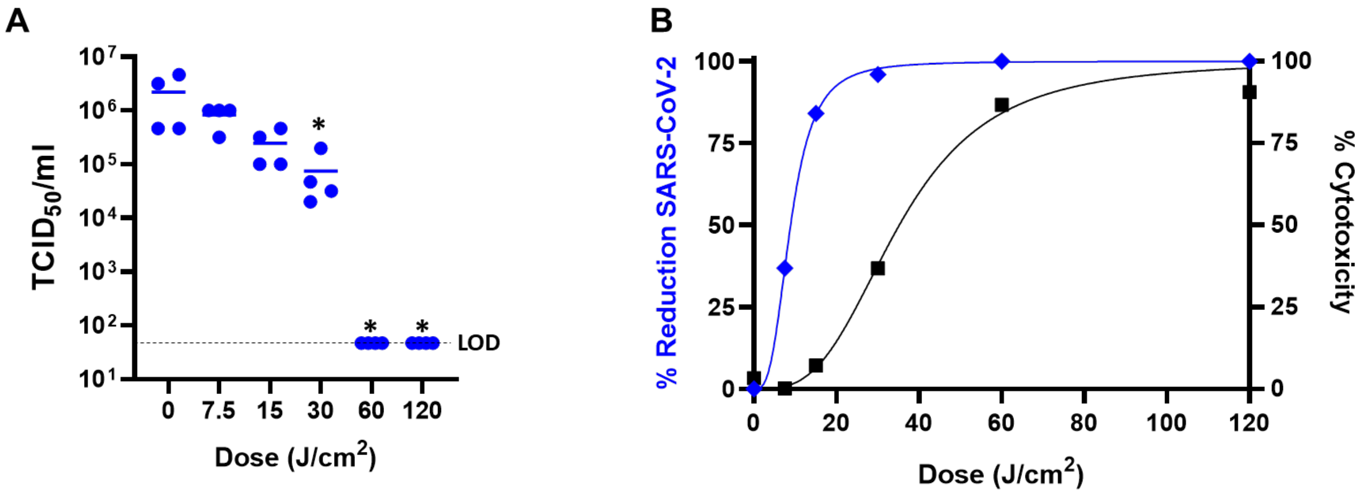

Supplementary Figure 3. 425 nm blue light inhibits SARS-CoV-2 replication in Vero 76 cells in a second, independent evaluation by laboratory 2. Vero 76 cells were infected with SARS-CoV-2 at a MOI of 0.01 and exposed to the indicated doses of 425nm light at 1-hour post-infection. (a) SARS-CoV-2 samples were harvested for $TCID_{50}$ assays at 48-hours post-infection (blue circles). The limit of detection (LOD) is indicated by the dashed line. (b) Cell viability was evaluated on Vero E6 that were not infected with virus and is represented as percent cytotoxicity (black squares/line). Percent reduction in SARS-CoV-2 virus was calculated at 48-hours post-infection (blue diamond/line, respectively). Data are represented as the mean of four independent measurements. Statistical analysis was executed using a Mann-Whitney test wherein * is $p < 0.05$ when compared to the 0 J/cm^2 control.

Supplementary Figure 4

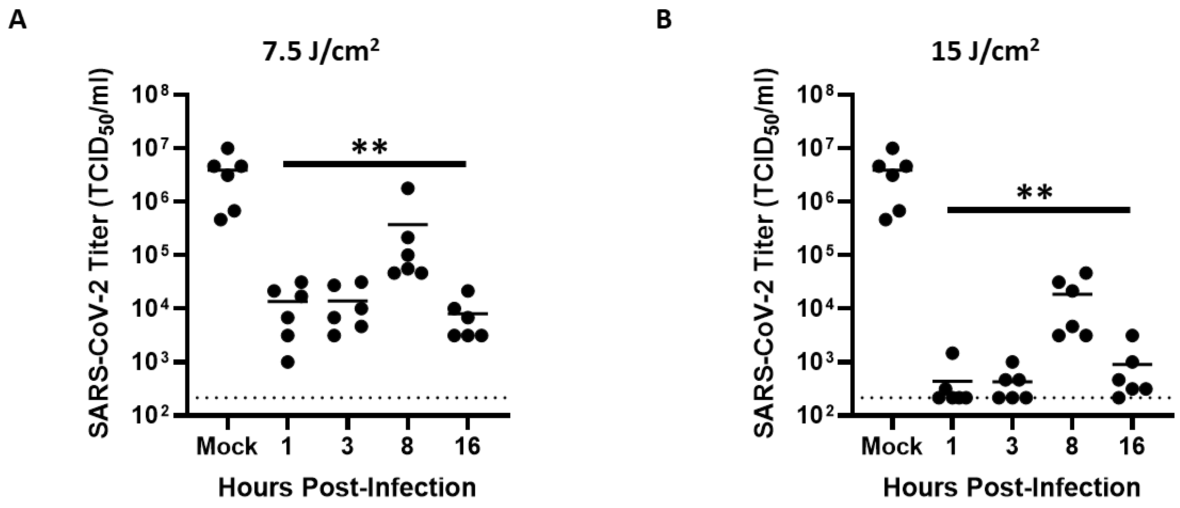

Supplementary Figure 4. 425 nm light inhibits SARS-CoV-2 replication following delivery at different times post-infection. Vero E6 cells were infected with SARS-CoV-2 and exposed to 7.5 J/cm^2 (a) or 15 J/cm^2 (b) of 425 nm light at different times post-infection. SARS-CoV-2 samples were harvested for $TCID_{50}$ assays at 24-hours post-infection (black circles, respectively). The limit of detection (LOD) is indicated by the dashed line. Data are represented as the mean of six independent measurements. Statistical analysis was executed using a Mann-Whitney test wherein ** is $p < 0.01$ when compared to the 0 J/cm^2 control.
